# HSV-1 Infection Differentially Modulates NPY and VIP Neuropeptide Expression in the Mouse Brain and in Human Neuronal Cells

**DOI:** 10.3390/ijms27156735

**Published:** 2026-07-28

**Authors:** Javier Carbone-Schellman, Nicolás Sales-Salinas, Rodrigo Reyes-Ramírez, Benjamín Rodríguez, Patricia Pereira-Sánchez, Gisella Vásquez-Canales, José A. Jara, Alfredo Molina-Berríos, Boris Rebolledo-Jaramillo, Alexis M. Kalergis, Pablo A. González, Luisa F. Duarte

**Affiliations:** 1 Centro de Medicina Regenerativa, Facultad de Medicina, Clínica Alemana, Universidad del Desarrollo, Santiago 7610717, Chile; javier.carbone@udd.cl (J.C.-S.); n.salessalinas@uandresbello.edu (N.S.-S.); benja.rodriguez.m@gmail.com (B.R.); 2 Millennium Institute on Immunology and Immunotherapy, Santiago 8331150, Chile; rodrigoreyesr1992@gmail.com (R.R.-R.); patricia.pereira@uc.cl (P.P.-S.); akalergis@uc.cl (A.M.K.); pagonzam@uc.cl (P.A.G.); 3 Facultad de Ciencias Biológicas, Pontificia Universidad Católica de Chile, Santiago 8330025, Chile; 4 Institute for Research in Dental Sciences, Faculty of Dentistry, Universidad de Chile, Santiago 8380544, Chile; gvasquez@odontologia.uchile.cl (G.V.-C.); jsandovalj@uchile.cl (J.A.J.); aemolina@u.uchile.cl (A.M.-B.); 5 Rare Diseases Program, Center for Genetics and Genomics, Institute of Science and Innovation in Medicine, Facultad de Medicina, Clínica Alemana, Universidad del Desarrollo, Santiago 7610717, Chile; brebolledo@udd.cl; 6 Departamento de Endocrinología, Facultad de Medicina, Pontificia Universidad Católica de Chile, Santiago 8330023, Chile

**Keywords:** herpes simplex encephalitis, neuropeptides, neuropeptide Y, vasoactive intestinal peptide, neuroimmunology, brain infection, BALB/c, C57BL/6, ICP34.5, neuroinflammation

## Abstract

Neurotropic viruses can alter neuronal responses in the central nervous system (CNS), significantly affecting viral clearance and disease progression. Herpes simplex virus type 1 (HSV-1) brain infection may lead to life-threatening severe acute encephalitis in untreated patients and neurological sequelae in survivors despite antiviral treatment. Notably, asymptomatic brain infection occurs in an important proportion of healthy individuals (>35%) and is associated with residual chronic neuroinflammatory responses that may lead to neurodegeneration. Therefore, understanding the molecular basis of these detrimental effects and finding and advancing new therapeutic strategies to manage HSV-1 brain infections are needed. Neuropeptides are pleiotropic neuroimmune mediators expressed throughout the CNS that modulate glial activation, cytokine production, and neuronal survival. However, their regulation during HSV-1 brain infections remains largely unexplored. Here, we sought to investigate the expression dynamics of two neuropeptides, neuropeptide Y (NPY) and vasoactive intestinal peptide (VIP), in two mouse strains that model human traits of symptomatic and asymptomatic HSV-1 brain infections (BALB/c and C57BL/6, respectively), as well as in the human neuroblastoma cell line SH-SY5Y, to uncover potential differences that could help explain the susceptibility of some individuals to develop severe HSV-1 infection. Our findings provide evidence that HSV-1 brain infection modulates NPY and VIP mRNA expression in a neurovirulence- and host-susceptibility-dependent manner, which may be associated with disease severity and chronic damage, warranting further evaluation.

## 1. Introduction

Herpes simplex virus type 1 (HSV-1) is a highly prevalent neurotropic virus that is primarily acquired during childhood [1]. It is estimated that approximately 67% of individuals under the age of 50 are infected, with the majority being asymptomatic [2]. Following primary infection of mucosal epithelial cells and peripheral sensory neurons, HSV-1 establishes lifelong latency in the trigeminal and dorsal root ganglia, with the capacity to periodically reactivate [1,3]. Occasional spread to the central nervous system (CNS) can result in herpes simplex encephalitis (HSE), an acute necrotizing condition with an estimated annual incidence of 1 per 250,000 to 500,000 individuals worldwide [4]. Importantly, asymptomatic HSV-1 brain infection has also been reported in about 35% of seropositive individuals without clinical signs of active infection or neurological illness, establishing a niche of viral persistence that could contribute to chronic neuroinflammation and detrimental effects, including neurocognitive impairment and cellular senescence [5,6].

Mouse models that recapitulate aspects of human brain infection have been essential for deepening our knowledge of the alterations induced by HSV-1 brain infection [7]. Indeed, several studies have reported BALB/c mice as an accurate model of HSE, as they develop severe acute viral encephalitis, exhibit severe neurological signs, show a high viral burden in the brain, and experience significant mortality [8,9,10]. Conversely, C57BL/6 mice are largely resistant to HSV-1 encephalitis, mainly due to a rapid and effective alpha/beta interferon (IFN-α/β)-mediated innate immune response, that limits viral pathogenesis, increases survival, and prevents neurological signs, mirroring scenarios in humans with asymptomatic HSV-1 brain infection [11,12]. Collectively, these two models constitute a powerful comparative framework for identifying host-intrinsic determinants of infection severity.

Neuropeptides are small endogenous signaling molecules produced and secreted mainly, but not exclusively, by neurons of the peripheral nervous system (PNS) and CNS [13]. These molecules have been recognized as important regulators of disease severity and progression into the CNS due to their extensive immunomodulatory effects [14,15,16,17]. Among these, neuropeptide Y (NPY) and vasoactive intestinal peptide (VIP) are particularly well-established as immunomodulatory molecules that regulate glial activation and cytokine production within the CNS. NPY is a 36-amino acid peptide widely distributed throughout the CNS [18], with recognized anti-inflammatory properties mediated through several receptor subtypes, primarily NPY1R, NPY2R, and NPY5R [19]. Likewise, VIP is a 28-amino acid neuropeptide belonging to the secretin/glucagon superfamily that signals mainly through the Vasoactive Intestinal Peptide Receptors 1 and 2 (VIPR1 and VIPR2) and, to a lesser extent, Pituitary Adenylate Cyclase-Activating Polypeptide Type I Receptor (PAC1R), inhibiting microglia activation and the production of TNF-α, IL-1β, IL-6, and nitric oxide via cAMP/PKA-dependent mechanisms [20,21]. Despite their reported roles in CNS immune homeostasis, whether NPY and VIP are differentially modulated during symptomatic and asymptomatic HSV-1 brain infection has not been previously investigated.

The aim of this study was to evaluate how HSV-1 brain infection modulates NPY and VIP expression in both susceptible (BALB/c) and resistant (C57BL/6) mouse strains during the acute and latent phases of infection. Furthermore, we assessed the effect of HSV-1 infection on the expression of these neuropeptides and their major receptors in a human neuroblastoma cell line (SH-SY5Y).

## 2. Results

### 2.1. Differential Susceptibility to HSV-1 Brain Infection in BALB/C and C57BL/6 Mice upon Intranasal Inoculation

To investigate whether HSV-1 infection modulates NPY and VIP expression in the brain across distinct severity outcomes, we first validated that our experimental models recapitulate the previously described symptomatic and asymptomatic outcomes [8,9,10,11,12]. BALB/c and C57BL/6 mice were intranasally infected with either the neurovirulent HSV-1 strain (F) or the neuro-attenuated ICP34.5-deletion mutant (Δ34.5), and we monitored the clinical progression by assessing body weight changes and clinical signs of HSE. The inclusion of the Δ34.5 mutant allowed us to determine the contribution of viral neurovirulence, as deletion of ICP 34.5 viral protein markedly impairs HSV-1 neuroinvasion, replication, latency establishment, and reactivation from the nervous system [22].

**Figure 1:**
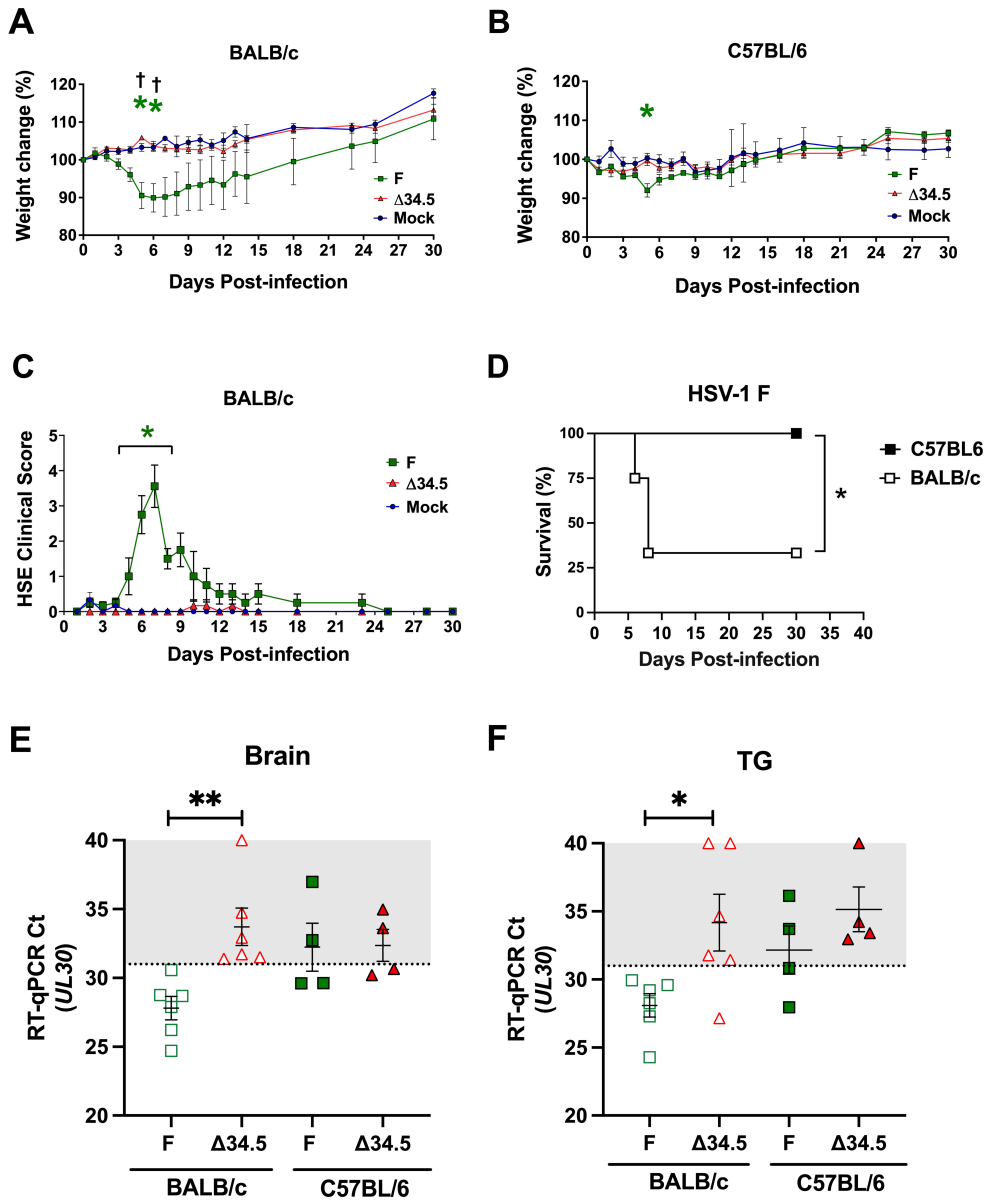
C57BL/6 mice exhibit resistance to HSV-1 infection, while BALB/c mice are highly susceptible. Female BALB/c and C57BL/6 mice were intranasally (i.n.) infected with wild-type (WT) HSV-1 (F, green line), a neuro-attenuated mutant strain (Δ34.5, red line), or mock-treated (blue line). Mice were monitored for 30 days. (**A**) Weight changes in BALB/c mice. Crosses indicate the time points at which animals were sacrificed as a human endpoint; * *p* < 0.05; †: deceased animal. (**B**) Weight changes in C57BL/6 mice. (**C**) Herpes simplex encephalitis (HSE) clinical scores for BALB/c mice. During the first 2 weeks post-infection, mice were clinically scored daily for neurological symptoms as follows: normal (0), ataxia (1), hunched posture (1), forelimbs paralyzed yet mobile-capable (1), forelimbs paralyzed and immobility (2), seizures or circling (1). The scores corresponding to each symptom are added and the final clinical score is accumulated. Data are presented as means ± SEM, pooled from two independent experiments. Statistical analysis was performed using two-way ANOVA followed by Dunnett’s multiple comparisons post-test; * *p* < 0.05. (**D**) Survival curve of C57BL/6 (n = 6) and BALB/c (n = 12) mice after i.n. infection with 2 × 10^5^ PFU of the F strain of WT HSV-1. Three BALB/c mice succumbed on day 5 and five more on day 6. Comparison of survival curves was evaluated by the log-rank (Mantel–Cox) test; * *p* < 0.05. HSV-1 RNA (RT-qPCR, *UL30* gene) in neural tissues: (**E**) brain and (**F**) trigeminal ganglia (TG) at day 4 post-infection. The Ct cutoff was determined with mock-inoculated samples. Statistical analysis was measured by one-way ANOVA with Bonferroni’s multiple comparisons post-test; ** *p* < 0.01, * *p* < 0.05.

As shown in Figure 1, BALB/c mice infected with the F strain exhibited significant and progressive body weight loss compared with mock-treated mice, whereas ∆34.5-infected mice did not show significant changes in body weight (Figure 1A). Importantly, eight animals in the F strain-infected group reached the humane endpoint (>20% body weight loss) and were euthanized on days 5–6 post-infection (d.p.i). By contrast, a slight weight loss was observed in the C57BL/6 mice infected with the F strain, which was statistically significant on day 5 post-infection compared with mock-treated mice. Δ34.5-infected C57BL/6 mice showed weight curves comparable to the mock controls (Figure 1B).

Regarding the HSE clinical score, BALB/c mice infected with the F strain showed significantly higher scores between four and eight d.p.i. than mock-treated mice (Figure 1C). As expected, neuro-attenuated ∆34.5-infected mice did not show signs of HSE (Figure 1C). Similarly, C57BL/6 mice did not show symptoms related to HSE regardless of the viral strain. Finally, the survival rates confirmed that BALB/c is a mouse strain susceptible to HSE, with only 33% of mice surviving F strain infection, compared with the established resistant C57BL/6 mouse strain, in which 100% survival was observed (Figure 1D).

Moreover, we evaluated the expression of the viral polymerase gene (*UL30*) in brain and trigeminal ganglia (TG) homogenates by RT-qPCR to detect the virus in neural tissues at 4 d.p.i. (Figure 1E,F). These results confirmed that HSV-1 reached the brain of all BALB/c mice infected with the F strain and that deletion of the ICP34.5 gene impaired its neuroinvasive capacity, as evidenced by significantly higher cycle threshold (Ct) values in ∆34.5-infected animals than in F-infected mice (Figure 1E). A similar pattern was observed in TG tissues of BALB/c mice (Figure 1F). In contrast, only half of the C57BL/6 mice showed detectable UL30 expression in the brain tissue for both viral strains, and the Ct values were consistently higher than those observed in F-infected BALB/c mice, suggesting a lower viral burden in the resistant strain (Figure 1E). Moreover, in TG, only two of four C57BL/6 animals in the F-infected group reached the positivity threshold, while all Δ34.5-infected animals remained below the detection threshold (Figure 1F).

Overall, these results validate our experimental approach by demonstrating two clearly distinct outcomes of HSV-1 brain infection, severe encephalitis in BALB/c mice and asymptomatic brain infection in C57BL/6 mice, with the disease outcome determined by both host and pathogen characteristics.

### 2.2. NPY and VIP mRNA Expression Were Differentially Regulated in the Brain During HSV-1 Infection, Depending on Viral Neurovirulence and the Phase of Infection

**Figure 2:**
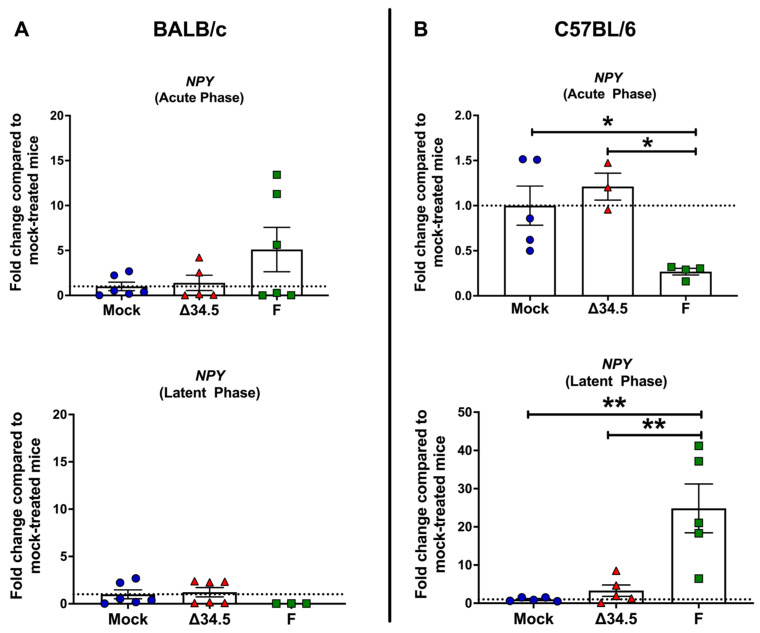
NPY mRNA expression in the resistant mouse strain shows significant changes over time and across levels of neurovirulence. Mice were mock-treated (blue circles) and intranasally (i.n) infected with neuro-attenuated F strain (Δ34.5, red triangles) and WT HSV-1 (F strain, green squares). NPY mRNA expression was assessed in bulk brain tissue on day 4 of (**A**) BALB/c or (**B**) C57BL/6 mice during the acute phase of infection (**upper panel**) and on day 30 when latency was established (**lower panel**). Values are presented as means ± SEM. Statistical analysis was performed using one-way ANOVA followed by Tukey’s post hoc test; ** *p* < 0.01, * *p* < 0.05.

Previous studies have reported that neurotropic viruses can alter neuropeptide expression in the brain during infection, thereby influencing viral clearance and disease progression [23,24]. We therefore investigate whether HSV-1 brain infection similarly modulates NPY and VIP expression *in vivo* and whether this response differs between the acute productive phase and the latent phase of infection. To address this question, NPY mRNA levels were first quantified by RT-qPCR in brain tissues collected from C57BL/6 and BALB/c mice infected with either F or ∆34.5 mutant virus or mock-inoculated at 4 and 30 d.p.i., corresponding to the acute and latent phases, respectively. BALB/c mice did not show an altered NPY expression pattern following acute HSV-1 infection (Figure 2A). Similarly, no significant modulation of NPY was detected during the latent phase in BALB/c mice that survived to day 30 of infection (Figure 2A).

In contrast, C57BL/6 mice display a dynamic modulation of NPY expression that depends on both the stage of infection and viral neurovirulence. During the acute phase, F virus infection showed a statistically significant decrease in mRNA levels compared with both mock-treated and ∆34.5-infected animals (Figure 2B). Interestingly, this pattern was reversed during the latent phase of infection, where F-infected animals showed significantly higher NPY mRNA levels than mock-treated and neuro-attenuated infected animals (Figure 2B). These findings indicate that NPY mRNA expression undergoes a biphasic regulation in the brains of animals that successfully controls HSV-1 infection (C57BL/6), characterized by early suppression followed by later upregulation that could depend in part on the viral neurovirulence.

**Figure 3:**
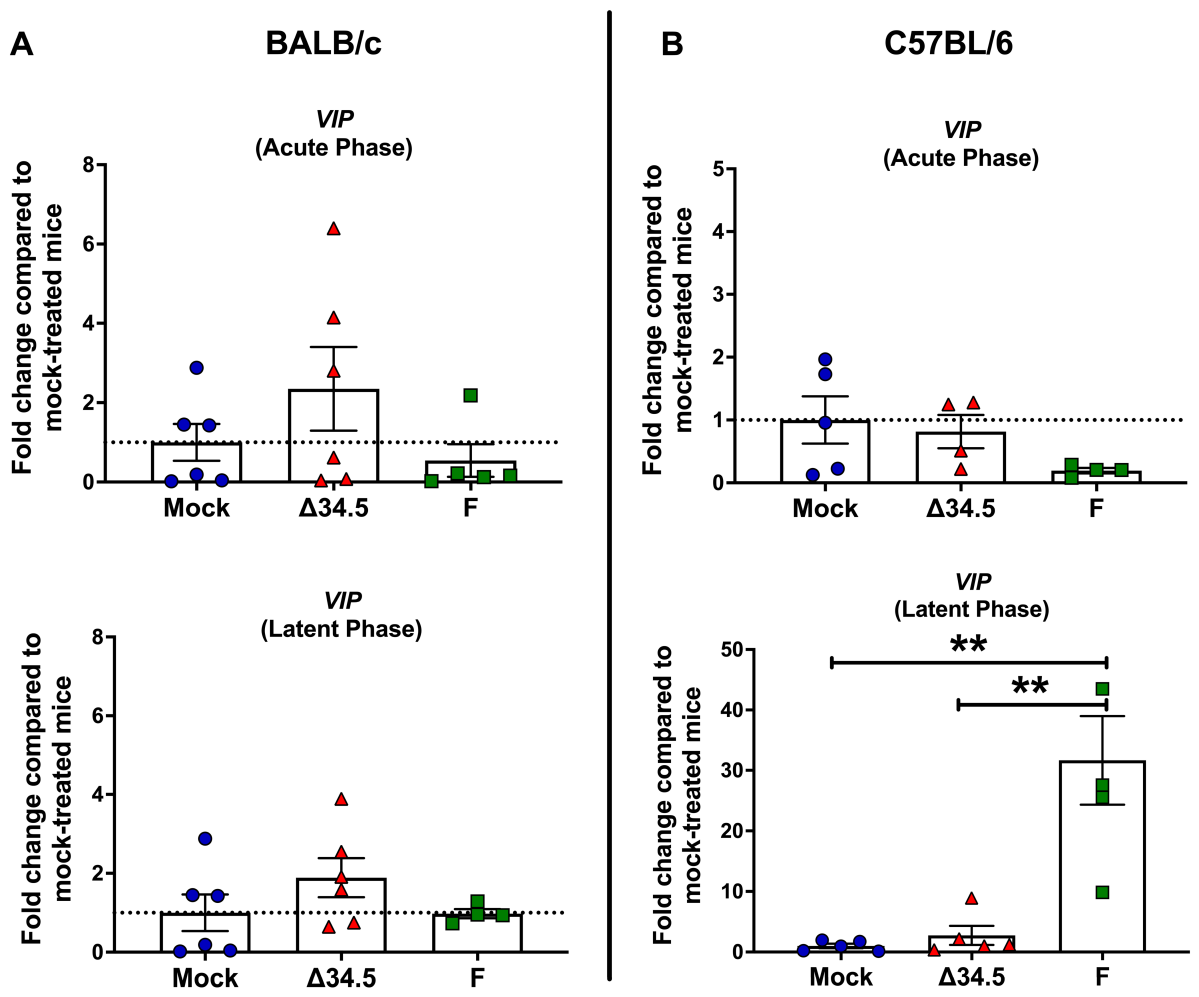
VIP mRNA expression is significantly increased in the HSV-1-resistant mouse strain during latency. (**A**) BALB/c mice and (**B**) C57BL/6 mice were mock-treated (blue circles) and intranasally (i.n.) infected with neuro-attenuated F strain (Δ34.5, red triangles) and WT HSV-1 (F strain, green squares). VIP mRNA expression was assessed in bulk brain tissue on day 4 during the acute phase of infection (**upper panel**) and on day 30 when latency was established (**lower panel**). Values are presented as means ± SEM. Statistical analysis was performed using one-way ANOVA followed by Tukey’s post hoc test (** *p* < 0.01).

Aside from NPY, VIP has emerged as another potent immunomodulatory peptide with pleiotropic roles that depend on the context [25]. To date, there is well-documented information about its anti-inflammatory and neuroprotective roles [26]. However, information about their role in viral infections is limited, and contradictory roles have been reported depending on the infection context [27,28]. To further understand the role of HSV-1 infection in VIP expression, VIP mRNA levels were also evaluated in the same brain samples collected from C57BL/6 and BALB/c mice by RT-qPCR. Similar to the results observed for NPY, the VIP mRNA levels did not show significant changes in the BALB/c mouse model, with comparable expression patterns among the three experimental groups across both the acute and latent phases (Figure 3A).

On the other hand, VIP mRNA levels showed a trend toward reduction during the acute phase of infection in the asymptomatic C57BL/6 mouse model compared to mock-treated and ∆34.5-infected animals. Importantly, this modulation did not reach statistical significance but showed strong consistency among animals infected with the neurovirulent strain (Figure 3B). As with NPY, the VIP mRNA levels showed a statistically significant upregulation in the latent phase of infection in this mouse model in the F-infected group compared to ∆34.5-infected and mock-inoculated groups (Figure 3B). This phenomenon reinforces the idea that neurovirulence plays an important role in the regulation of the expression of these neuropeptides. Moreover, the biphasic pattern of expression during the acute and latent phases in the resistant mouse strain could reflect the host’s need to induce a protective pro-inflammatory response at the onset of infection and subsequently resolve inflammation to reduce excessive collateral damage, warranting further evaluation.

To place our findings in the context of independent data, we reanalyzed the publicly available brainstem RNA-seq dataset GSE168799 [29], which compared female wild-type (wt Rel^+/+^) and c-Rel-mutant (Rel^C307X^) C57BL/6 mice under non-infected and HSV-1-infected conditions, the latter susceptible to HSE. Infected animals were further stratified by viral load as low or high responders. Across all the comparisons examined, neither NPY nor VIP reached significance after multiple-testing correction (*p* adjusted values (*padj*) > 0.05; Supplementary Figure S1, Supplementary Table S1). The only comparison in which differential gene expression reached nominal significance was NPY between high- and low-viral-load mutant mice (nominal *p* = 0.0083), where a higher viral load was associated with lower NPY expression. Although this association did not remain significant after multiple-testing correction (*padj* = 0.2275), this trend aligns with our observation that NPY mRNA levels were significantly reduced in C57BL/6 mice infected with the neurovirulent HSV-1 strain compared to those infected with the neuro-attenuated ∆34.5 mutant in the acute phase, suggesting that NPY downregulation may depend on the magnitude of viral replication in this mouse strain.

### 2.3. NPY and VIP mRNA Expression Show Strain-Dependent Differences During the Latent Phase of Survivor Mice

**Figure 4:**
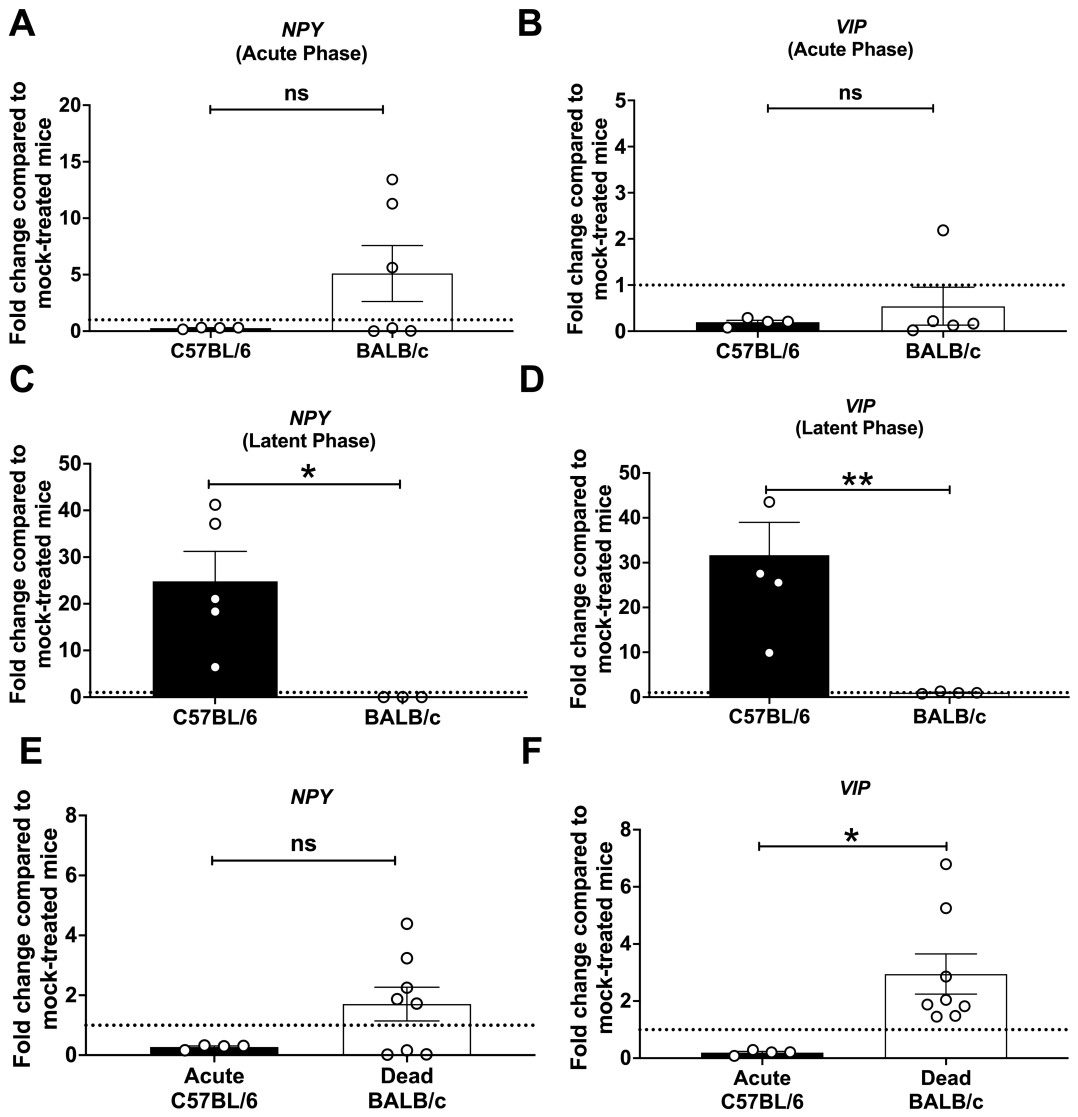
NPY and VIP mRNA levels are elevated in response to severe acute BALB/c infection but do not reach the levels induced by C57BL/6 at later time points. (**A**,**B**) Comparison of NPY (**A**) and VIP (**B**) mRNA levels in brain homogenates from C57BL/6 and BALB/c mice infected with neurovirulent HSV-1 (F strain) and sacrificed at 4 days post-infection (d.p.i.), representing the acute phase. (**C**,**D**) Comparison of NPY (**C**) and VIP (**D**) mRNA levels in brain homogenates from C57BL/6 and BALB/c mice obtained at 30 d.p.i., representing the latent phase. (**E**,**F**) Comparison of NPY (**E**) and VIP (**F**) mRNA levels between C57BL/6 mice sampled during the acute phase and BALB/c mice that succumbed to HSV-1 infection during the acute phase. The datasets used in these analyses are from the same animals shown in Figure 2 and Figure 3. Data represent mean ± SEM. Dotted lines indicate the reference level of mock-treated mice. Statistical comparisons were performed using an unpaired Student’s *t*-test. * *p* < 0.05, ** *p* < 0.01, ns = non-significant.

To further determine whether neuropeptide expression is influenced by host susceptibility and differs significantly between asymptomatic and symptomatic models of HSV-1 brain infection, we compared the NPY and VIP mRNA levels in both mouse strains during the acute and latent phases of infection using the same dataset. No significant differences in the mRNA levels of both neuropeptides were detected between the two strains during the acute phase (Figure 4A,B). In contrast, during the latent phase (30 days post-infection), both NPY and VIP mRNA levels were significantly upregulated in C57BL/6 mice compared with the BALB/c mice that survived infection (Figure 4C,D).

Given these differences in neuropeptide expression between susceptible and resistant mouse strains, we sought to determine whether low NPY and VIP mRNA levels in BALB/c mice were associated with poorer clinical scores and higher mortality. To do so, we compared the expression of both neuropeptides in BALB/c mice that succumbed to infection during the acute phase with that observed in C57BL/6 mice at a similar time point. Surprisingly, BALB/c mice that died during the acute phase exhibited higher mRNA levels of both neuropeptides than C57BL/6 mice at 4 days post-infection, although this difference reached statistical significance only for VIP (Figure 4E,F). However, when the NPY and VIP protein levels were evaluated in brain homogenates by Western blot, only a trend toward increased expression was observed in BALB/c mice, with no statistically significant differences compared with C57BL/6 animals (Supplementary Figure S2). It is also important to note that BALB/c mice never reached the markedly elevated mRNA levels observed in C57BL/6 mice during the latent phase. These findings suggest a reactive yet insufficient upregulation of these neuropeptides in a susceptible host and that a threshold level of NPY and VIP expression in the brain may be required for protection against lethal HSV-1 encephalitis. However, further mechanistic studies are needed to determine whether NPY and VIP play a regulatory role during HSV-1 brain infection.

### 2.4. HSV-1 Infection Upregulates NPY and VIP Expression in Human Neurons and Downregulates NPY1R in a Virus Replication-Dependent Manner

**Figure 5:**
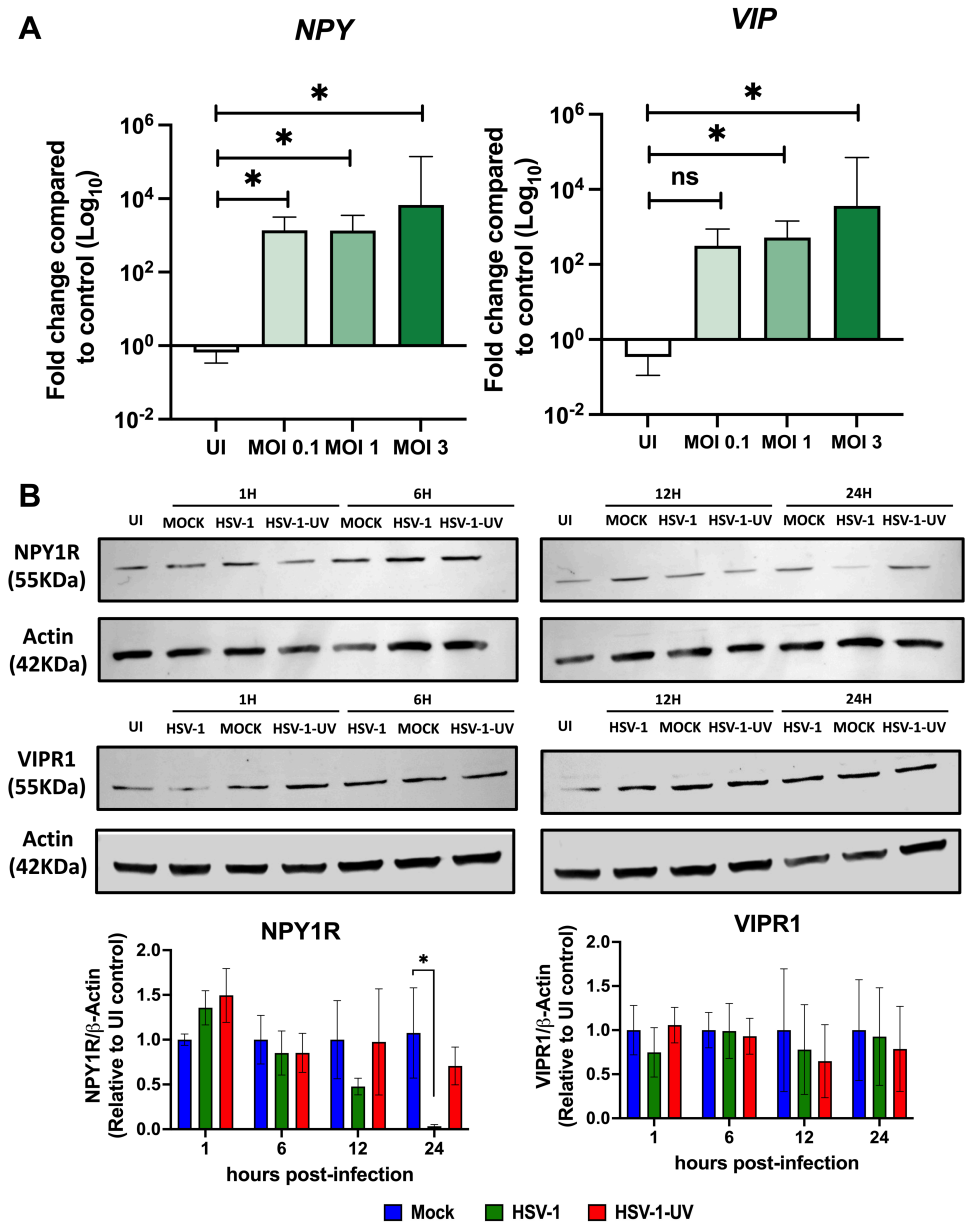
HSV-1 infection upregulates mRNA levels of neuropeptides and downregulates protein levels of the NPY1 receptor in human SH-SY5Y neurons. (**A**) RT-qPCR analysis of NPY (**left**) and VIP (**right**) mRNA levels at increasing MOI (0.1, 1, 3) relative to uninfected controls (UI) in SH-SY5Y cells; values are shown in log10 scale, as mean ± SEM. Statistical analysis was measured by one-way ANOVA with Dunnett’s multiple comparisons post-test; * *p* < 0.05, ns = non-significant. (**B**) Representative Western blots of NPY1R (**left**) and VIPR1 (**right**) at 1, 6, 12, and 24 h.p.i. of mock-treated, HSV-1-F, and HSV-1-F-UV infected SH-SY5Y cells; β-Actin was used as a loading control. Graphs show the densitometric quantification of NPY1R and VIPR1 protein levels, normalized to uninfected controls; mean ± SEM. Two ANOVA with Dunnett’s multiple comparison post hoc test against mock group at each time point; * *p* < 0.05.

Given the findings obtained in the mouse models, we next investigated whether HSV-1 infection modulates neuropeptide expression in human neuronal cells. To assess this, we infected SH-SY5Y human neuroblastoma cells with the HSV-1-F strain at increasing multiplicities of infection (MOI) of 0.1, 1, and 3, and the NPY and VIP mRNA expression was quantified by RT-qPCR. We observed that the NPY mRNA levels were significantly elevated after infection at all MOIs evaluated, whereas the VIP mRNA levels showed a significant increase at MOI 1 and MOI 3 compared with uninfected cells (Figure 5A). Moreover, we evaluated whether HSV-1 infection also affected the expression of the principal receptors that display the highest binding affinity for NPY and VIP, NPY1R and VIPR1, by Western blot analysis. Protein levels were evaluated at 1, 6, 12, and 24 h.p.i., using an MOI of 1. Additionally, to determine whether the observed modulation depends on viral replication in neurons, parallel cells were infected with ultraviolet (UV)-inactivated F virus (HSV-1-UV) as a replication-deficient control.

Interestingly, the NPY1R protein levels were significantly reduced at 24 h.p.i. in cells infected with the F strain compared to mock-inoculated controls, an effect that was absent in cells exposed to UV-HSV-1, indicating a replication-dependent effect (Figure 5B). In contrast, VIPR1 protein levels remained unchanged throughout all the time points evaluated, regardless of the viral condition (Figure 5B). Overall, these results show that HSV-1 infection of human neuronal cells induces a replication-dependent upregulation of NPY and VIP mRNA and further induces a decrease in NPY1R protein levels.

We next evaluated whether this alteration in NPY1R protein expression could also be observed *in vivo*. Western blot analysis of brain homogenates at 4 d.p.i. from BALB/c and C57BL/6 mice infected with either the neurovirulent F strain or the neuro-attenuated Δ34.5 mutant did not show significant differences in NPY1R protein levels compared with mock-infected controls (Supplementary Figure S3). These findings suggest that, unlike the changes observed in cultured human neurons, NPY1R expression remains largely unchanged at the whole-brain level during HSV-1 infection, possibly reflecting cell type-specific regulation that cannot be detected in tissue homogenates (Supplementary Figure S3).

Finally, we sought to determine whether NPY or VIP could inhibit HSV-1 replication in neurons. We performed a time-of-addition assay, in which SH-SY5Y cells were pre- or post-treated with NPY, VIP, or a VIPR1 antagonist, added exogenously at different non-toxic concentrations (Supplementary Figure S4). However, we did not observe a significant change in the number of viral particles released after 24 h post-infection in any of the evaluated conditions in comparison with the untreated control (Supplementary Figure S5). This contrasted with acyclovir-treated SH-SY5Y cells, which displayed a significant decrease in the average of plaque-forming units count and viral protein expression, as assessed by plaque and Western blot assays, respectively (Supplementary Figure S5). Taken together, these results suggest that these neuropeptides are not exerting a direct antiviral effect on neurons. It remains to be evaluated whether they exert a paracrine effect on neighboring cells or a neuroimmunomodulatory role *in vivo*, rather than an antiviral effect.

## 3. Discussion

Given the dual clinical outcomes of HSV-1 infection, in which approximately 80% of infected individuals are asymptomatic, and a smaller fraction experiences severe diseases such as HSE or herpetic keratitis [30,31,32], the use of animal models that reliably recapitulate these distinct clinical scenarios is essential for identifying the mechanisms that could, at least in part, explain natural resistance to HSV-1 infection or susceptibility to severe disease. Previous studies have reported that the disease outcome after HSV-1 infection is influenced by multiple host and viral factors, including the route of infection, type-I interferon response, the IFN regulatory factor 3 (IRF3) signaling pathway, the viral strain, the mouse strain, and inoculum dose [33,34,35]. For instance, whereas infection with the Rodanus strain produces lesions confined to the trigeminal root entry zone (TREZ) in both Swiss and BALB/c mice [11], infection with the F strain produces demyelination in both the TREZ and the brainstem (BST) of the two mouse strains [11]. In contrast, the HSV-1 McKrae strain fails to induce CNS demyelination in either BALB/c or C57BL/6 mice unless macrophages are experimentally depleted, highlighting the critical role of the host immune response in disease development [36]. Likewise, it has been reported that differential susceptibility between inbred mouse strains results primarily from the host’ s capacity to mount rapid IFN-α/β responses that limit viral dissemination into the nervous system, rather than from intrinsic differences in viral neuroinvasion [8]. In our intranasal infection model, BALB/c mice were susceptible to brain infection and exhibited severe clinical pathology, whereas C57BL/6 mice achieved effective viral control within the nervous system without evident clinical symptoms of encephalitis. Moreover, the ICP 34.5-deleted mutant (Δ34.5) proved a suitable tool for dissecting the contribution of viral neurovirulence. Notably, the ICP 34.5 HSV-1 protein inhibits type-I IFN, autophagy, and host-mediated shut-off of protein synthesis in order to evade the innate host immune response, which is required for viral dissemination and HSV-1-related pathogenesis in the brain [37,38]. We observed that both mouse strains infected with this neuro-attenuated virus exhibited asymptomatic infection. Importantly, despite extensive recognition of several host and pathogen determinants in HSV-1 CNS pathology, the contribution of neuroimmune modulators, such as the neuropeptides NPY and VIP, has not been previously evaluated.

Neuropeptides are pleiotropic neuroimmune molecules widely expressed throughout the CNS that modulate glial activation, cytokine production, and neuronal survival [15,17,39]. Here, we investigated whether HSV-1 brain infection differentially modulates the expression of NPY and VIP in two well-established mouse models that recapitulate symptomatic (BALB/c) and asymptomatic (C57BL/6) HSV-1 brain infection, as observed in humans. Our results revealed a biphasic neuropeptide response in C57BL/6 mice, with downregulation during the acute phase and upregulation during the latent phase, suggesting that viral suppression of anti-inflammatory mediators early after infection may be part of an immune-evasion strategy. Alternatively, given that both VIP and NPY inhibit TNF-α, IL-1β, and NF-κB [40,41,42], which are key molecules of antiviral pathways, their downregulation during the peak of viral replication could transiently amplify the proinflammatory milieu needed for effective viral clearance. On the other hand, it has been well documented that latent HSV-1 brain infection is associated with chronic neuroinflammation, in which microglia remain activated in the brains of HSV-1-infected mice at 30 days post-infection, along with persistent immune cell infiltration, which further contributes to neuronal damage and the accumulation of neurodegenerative markers [6,43,44,45]. In this context, we speculated that the upregulation of NPY and VIP in C57BL/6 mice at day 30 may represent a compensatory neuroprotective response, which could be associated with dampening sustained glial activation and limiting immunopathological damage. However, whether this upregulation is functionally neuroprotective remains to be determined. Moreover, it is important to note that neuropeptide expression in C57BL/6 mice was analyzed, including animals with detectable or undetectable *UL30* expression in neural tissues, as neuroinflammatory consequences of primary HSV-1 infection have been documented to persist beyond the clearance of detectable virus and may therefore influence neuropeptide expression independently of active viral replication. Nevertheless, we acknowledge that neuropeptide levels may differ between animals with confirmed CNS infection and those without detectable viral RNA, and future studies with larger cohorts should include stratified analyses to address this. Notably, we reanalyzed a publicly available brainstem RNA-seq dataset (GSE168799) from C57BL/6 female *Rel^C307X^* mutant mice, which are susceptible to HSE, and from resistant WT *Rel*^+*/*+^ littermates collected at day 5 post-infection (Supplementary Figure S1) [29]. This analysis showed partial agreement with our results, as neither NPY nor VIP expression differed between susceptible and non-infected animals. However, unlike our whole-brain RT-qPCR analysis, no acute reduction of NPY was observed in wild-type mice compared to uninfected mice. This discrepancy may be explained, at least in part, by lower infectious dose used in the RNA-seq study, which may have resulted in a reduced viral burden during the acute phase. Supporting this interpretation, a decreased NPY expression with a significant nominal *p*-value was only detected in susceptible mutant mice with high viral loads compared to those with low viral loads, suggesting that NPY downregulation may be associated with the magnitude of viral replication in this mouse strain. This observation is consistent with our findings, in which a significant decrease in NPY mRNA expression was observed in mice infected with the neurovirulent HSV-1 strain in comparison to animals infected with the neuro-attenuated strain. Nevertheless, comparisons between studies should be interpreted with caution because of important methodological differences, including the brain region analyzed (whole-brain vs. brainstem), mouse model (naturally susceptible BALB/c strain vs. targeted genetic mutation in a naturally resistant C57BL/6 background), viral strain (F vs. 17syn+ HSV-1), inoculum (200.000 PFU vs. 50.000 PFU), and infection stage (acute and latent phases vs. only acute phase), all of which may influence neuropeptide expression.

Furthermore, the fact that the expression levels of the neuropeptides NPY and VIP remain unaltered in animals infected with a neuro-attenuated HSV-1 strain suggests that neurovirulence directly affects their expression. This interpretation is consistent with emerging evidence implicating NPY in the host response to neurotropic viral infections. In a mouse model of retroviral infection, NPY mRNA expression in the brain was upregulated and associated with viral neurovirulence. NPY-deficient (NPY^−/−^) mice developed neurological disease more rapidly than wild-type mice, with increased monocyte infiltration into the CNS [24,46]. This effect was partially rescued by treatment with an NPY2R-specific analog, indicating a protective role for NPY through NPY2R signaling [46].

Moreover, several studies indicate that neuropeptides can affect the outcome and severity of viral infections in a virus-specific and disease-context-dependent manner [24,46,47,48,49]. VIP has a context-dependent dual role in antiviral immunity. VIP-knockout mice showed enhanced cellular immune responses and better survival following murine cytomegalovirus (mCMV) infection, suggesting that VIP-mediated immunosuppression may impair viral clearance in this herpesvirus model [50]. By contrast, VIP and pituitary adenylate cyclase-activating polypeptide (PACAP) have been shown to reduce viral replication in HIV-infected macrophages and SARS-CoV-2-infected lung cells [47,49]. Moreover, the latter study found that plasma VIP levels were significantly higher in severe COVID-19 patients, correlated with survival, and functioned as a protective mechanism [49]. Additionally, VIP has been reported to reduce HIV-1 gp120-induced neuronal death in primary rat cortical neurons [51]. Here, we observed that VIP mRNA levels increased in BALB/c mice that died after HSV-1 infection. Whether VIP upregulation in these animals is associated with a deleterious role that led to poor outcomes, or whether this neuropeptide is transiently upregulated early after infection as part of a reactive anti-inflammatory response aimed at limiting neuronal damage and restoring immune homeostasis in the context of severe brain inflammation, remains to be evaluated. It is important to note that the differences in NPY and VIP expression observed at the mRNA level between BALB/c mice that succumbed to infection and acutely infected C57BL/6 mice were not mirrored at the protein level, as Western blot analysis of whole-brain homogenates revealed only a non-significant trend toward higher neuropeptide levels in BALB/c mice (Supplementary Figure S2). This apparent discrepancy between transcript and protein abundance is not unexpected and may reflect both technical and biological factors. First, RT-qPCR is more sensitive than Western blot assays for detecting relatively small changes in gene expression. Second, neuropeptides such as NPY and VIP undergo post-translational processing and are rapidly secreted after synthesis. Consequently, protein levels measured in tissue homogenates may underestimate biologically relevant changes in neuropeptide production [52]. Moreover, because both neuropeptides are actively released into the extracellular space, alterations in their secretion may be more accurately reflected in cerebrospinal fluid (CSF) or plasma than in brain homogenates. Future studies employing more sensitive quantitative approaches, such as the enzyme-linked immunosorbent assay (ELISA) or the Meso Scale Discovery (MSD) platform, to measure soluble neuropeptide levels in CSF and plasma will help clarify the role of these neuropeptides during HSV-1 CNS infection, as previously demonstrated in other viral infections [53,54].

Additionally, further research is needed to determine whether the magnitude of this neuropeptide response in BALB/c mice is insufficient to reach the threshold required for effective immunomodulation, as the mRNA levels of these neuropeptides were significantly higher in C57BL/6 mice at later time points. Moreover, we hypothesized that neuropeptide overexpression during the latent phase of HSV-1 infection could play a neuroprotective role against viral reactivation and chronic inflammation, possibly exerting a positive role for these neuropeptides during chronic HSV-1 infection. By contrast, it could impair antiviral immune surveillance, potentially facilitating viral persistence or reactivation, as in CMV infection, which remains to be determined.

Finally, in the human neuroblastoma SH-SY5Y cell line, HSV-1 infection induced significant upregulation of both NPY and VIP mRNA and drove a replication-dependent downregulation of NPY1R protein levels. We focused on NPY1R and VIPR1, because they exhibit the highest affinity for NPY and VIP, respectively, and represent the principal receptors mediating the immunomodulatory actions of these neuropeptides within the CNS [41,55]. Through NPY1R signaling, NPY suppresses NF-κB activation, nitric oxide production, and the release of pro-inflammatory cytokines such as IL-1β and IL-6, while also limiting microglial activation and motility [41], effects that are not replicated by NPY2R or NPY5R activation [56,57]. Similarly, VIP signaling through VIPR1 inhibits NF-κB activation and the production of multiple inflammatory mediators, including TNF-α, IL-6, IL-12, and nitric oxide, while promoting IL-10 production through cAMP-dependent mechanisms [55,58,59]. Notably, HSV-1 infection downregulates NPY1R protein levels at 24 h post-infection, which could affect NPY’s anti-inflammatory properties. This downregulation could render neurons less responsive to NPY’s neuroprotective signals. However, whether this decrease is replicated in other cell types, or whether it also affects other receptors of the same family, remains to be evaluated. Notably, this reduction in NPY1R protein was not detected in whole-brain homogenates from acutely infected BALB/c or C57BL/6 mice, where the receptor expression remained unchanged regardless of the infection status (Supplementary Figure S3). This discordance may reflect important differences in the cellular and molecular context between a homogeneous human neuroblastoma cell line and the complex multicellular composition of brain samples. In bulk brain homogenates, the NPY1R protein is expressed across multiple cell populations, including neurons, astrocytes, and microglia, that likely respond differently to infection, with changes in one cell type potentially being masked by stable or opposing expression in others. In addition, antiviral activity assays performed in SH-SY5Y cells showed that neither NPY nor VIP or the pharmacological blockade of VIP receptors exerted a direct antiviral effect against HSV-1 infection in neurons, as no significant reduction in viral replication or viral protein expression was observed upon treatment (Supplementary Figure S5). It will be important for future studies to determine whether they influence HSV-1 neuropathogenesis indirectly through paracrine signaling or by shaping the neuroimmune microenvironment *in vivo*.

The present study has several limitations that should be acknowledged to suggest future directions in the field. First, this work presents an initial characterization of NPY and VIP modulation, mainly at the mRNA level, during HSV-1 brain infection. It would be essential to evaluate the expression of these neuropeptides in CSF and plasma samples to assess their protein levels and release. Another important limitation of this study is the high mortality rate in BALB/c mice infected with wild-type HSV-1F virus, given the possibility that survivors differ from deceased animals in their immune response to infection, potentially representing a biologically selected subgroup. These differences in the host response among mice within the same strain are critical for understanding the key players in the antiviral response. This remains a clear gap in the field that remains to be investigated. Future studies including larger cohorts and intermediate sampling time points during the period of highest mortality will be important to define the temporal dynamics of neuropeptide expression across the full spectrum of disease severity. Moreover, additional functional experiments should be performed to establish a causal role for these neuropeptides in viral replication, neuroinflammation, neuronal survival, or disease outcome. This study lays the foundations for future studies aiming to evaluate their active contribution to the regulation of the neuroinflammatory response during CNS viral infections, such as mechanistic dissection by using exogenous neuropeptide treatment at different cell types or animal models, receptor-specific antagonists, and knockout animal models, as well as knockout or CRISPR-edited neuronal and brain resident cell lines, such as microglia. These approaches will enable the direct testing of whether the expression changes reported here have functional consequences.

## 4. Materials and Methods

### 4.1. Animals

Female C57BL/6 and BALB/c mice, aged 6 to 7 weeks, were housed at the central animal facility of Pontificia Universidad Católica de Chile. Both strains received environmental enrichment, sterile food, and water ad libitum. The study protocols were approved by the Scientific Ethical Committee for Animal and Environmental Care of Pontificia Universidad Católica de Chile (Protocol #230330016) and by the institutional Biosafety Committee of Pontificia Universidad Católica de Chile and Universidad del Desarrollo. All procedures were conducted in accordance with the National Institutes of Health Guide for the Care and Use of Animals [60].

### 4.2. Virus Propagation

Vero cells (ATCC CCL-81) were used to propagate and titrate the wild-type (WT) neurovirulent HSV-1 (strain F) and the neuro-attenuated ICP34.5-deficient HSV-1 F strain (∆34.5) for animal infection. T175 flasks containing Vero cell monolayers were inoculated with both strains at a multiplicity of infection (MOI) of 0.01 in 20 mL of Opti-MEM (Gibco, Life Technologies, Grand Island, NY, USA) and incubated at 37 °C for 48 h until a clear cytopathic effect was visible. The contents of the flasks were pooled, and cell debris was removed by centrifugation at 15,000× *g* for 10 min. The pellet was resuspended in 1 mL of Opti-MEM and then sonicated in an ultrasonic bath for 10 min with 10 s pulses and ice rest of 50 s between pulses. These stocks were aliquoted and stored at −80 °C until required.

To propagate the virus for *in vitro* assays, T175 flasks containing Vero cell monolayers were inoculated with the HSV-1 F strain at an MOI of 0.01 in 13 mL of Opti-MEM (Gibco, Life Technologies) and incubated at 37 °C for 72 h until a clear cytopathic effect was visible. The contents of the flasks were pooled and sonicated in an ultrasonic bath for 10 min with 10 s pulses and ice rests between pulses. Cell debris was removed by centrifugation at 5000 rpm for 10 min, and the stocks were aliquoted and stored at −80 °C until required. For the UV-attenuated virus, the HSV-1 F strain was exposed to 12 h of direct UV light.

For mock preparation, the same protocols were followed without virus inoculation, yielding a non-infectious supernatant from Vero cells.

### 4.3. Mouse Infections

Female mice were intranasally infected with 2 × 10^5^ plaque-forming units (PFU) of each viral strain (F and ∆34.5) in 10 µL of phosphate-buffered saline (PBS) after intraperitoneal anesthesia with Ketamine (80 mg/kg) and Xylazine (4 mg/kg). A non-infectious supernatant (mock) was used for healthy control animals, following the same protocol. During the first two weeks after infection, the animals were monitored daily for body weight, physiological parameters, and evaluation of the herpes simplex encephalitis (HSE) score. Physiological score symptoms included weight loss, appearance, hydration, and spontaneous behavior. The HSE score evaluation considered other aspects, such as ataxia, loss of balance, and coordination. After the initial two weeks, the animals were monitored 3 times a week. Scores were aggregated to provide representative clinical scores for physiological and encephalitis symptoms. The sample sizes were as follows: BALB/c mice: F (n = 6), Δ34.5 (n = 6) for evaluating parameters at 4 days post-infection, and F (n = 12), Δ34.5 (n = 6) for evaluating parameters at 30 days post-infection, of which 8 mice in the F-infected group succumbed to infection, and 4 mice survived until day 30. C57BL/6 mice: F (n = 4), Δ34.5 (n = 4) for evaluating parameters at 4 days post-infection, and F (n = 6), Δ34.5 (n = 6) for evaluating parameters at 30 days post-infection. Moreover, the mock-inoculated control groups comprised 6 mice per mouse strain.

### 4.4. In Vitro Experiments Evaluating the Expression of VIP, NPY, and Their Main Receptor

SH-SY5Y (ATCC, CRL-2266) cells were grown in Dulbecco’s Modified Eagle Medium (DMEM) supplemented with 5% fetal bovine serum (FBS; HyClone, Logan, UT, USA, CAT #SH30396.03HI) and 1% penicillin/streptomycin; then, they were incubated at 37 °C with 5% CO_2_ saturation to evaluate the NPY and VIP mRNA expression. We seeded cells in 6-well plates until reaching 80% confluence. Cells were infected with the desired MOI in OPTI-MEM and incubated for 1 h to allow viral adsorption. Following this, the remaining virus was replaced with Opti-MEM, and the cells were incubated for 24 h under the previously mentioned conditions. After incubation, cell pellets were resuspended in TRIzol reagent (Thermo Fisher Scientific, Waltham, MA, USA; CAT #15596026) for RNA extraction. To evaluate the NPY1R and VIPR1 protein levels following infection, cell pellets were lysed at 1, 6, 12, and 24 h post-infection for protein extraction. Attenuated virus (HSV-1 exposed to UV-light; UV-HSV-1) and mock were added as controls at each time point.

### 4.5. Cytotoxicity Assay

SH-SY5Y cells were seeded into 96-well plates at a density of 1.5–2 × 10^5^ cells/well and incubated with VIP (Tocris Bioscience, Bristol, UK, CAT #1911) and NPY (Tocris Bioscience, CAT # 1153) at concentrations of 0.1, 1, and 10 nM and µM, respectively, as previously reported [61,62]. Cells were also treated with the VIP receptor antagonist [D-p-Cl-Phe6,Leu17]-VIP (ANT; Tocris Bioscience, Cat. #3054) at concentrations of 10, 100, and 1000 nM, as previously reported [49,59]. All treatments were applied for 24 h at 37 °C in a humidified incubator with 5% CO_2_. Following treatment, the culture medium was removed, and the cell viability was assessed using a resazurin reduction assay (AlamarBlue™, Invitrogen, Carlsbad, CA, USA, CAT #DAL1025). Briefly, the reagent was diluted 1:10 in Opti-MEM, and 100 µL of the resulting solution was added to each well. Plates were incubated for 2 h at 37 °C, after which the fluorescence was measured at excitation/emission wavelengths of 560/590 nm. As a positive control for cytotoxicity, cells were exposed to 75% ethanol for 20 min prior to viability assessment under the same experimental conditions. Three independent experiments were performed for each treatment.

### 4.6. Time-of-Addition Assay

The effects of NPY, VIP, and VIP receptor antagonism on HSV-1 replication were evaluated using pre- and post-treatment experimental schemes by quantifying released infectious viral particles. SH-SY5Y cells were seeded in 96-well plates at a density of 1.5–2 × 10^5^ cells/well and treated with 100 µL of NPY (10, 5, 2.5 and 1.25 µM), VIP (50, 10, 5 and 1 ņM), ANT (1000, 100, 10 and 1 ņM), or acyclovir (ACV) (250 µg/mL). In the pre-treatment scheme, cells were incubated with the corresponding compounds for 24 h at 37 °C in a humidified atmosphere containing 5% CO_2_ and subsequently infected with 100 µL of HSV-1 at an MOI of 1 in the presence of the treatment for 1 h. Following viral adsorption, the inoculum and treatments were removed, and the cells were maintained in Opti-MEM medium for an additional 24 h. In the post-treatment scheme, cells were first infected with the virus for 1 h. The viral inoculum was then removed and replaced with the corresponding treatment solutions, and the cells were incubated for an additional 24 h under standard culture conditions. In both experimental schemes, culture supernatants were collected at 24 h.p.i. and stored at −80 °C until further analysis. The production of infectious viral particles was quantified by a plaque-forming unit assay on the Vero cell monolayer, in at least three independent experiments, and stained with 1% Crystal Violet for representative images. Moreover, the effects of VIP and ANT on the expression of viral glycoprotein B (gB) were evaluated independently by Western blot analysis. SH-SY5Y cells were seeded in 6-well plates at a density of 2–3 × 10^6^ cells/well. A mock-infected group served as a negative control, whereas ACV-treated cells subjected solely to the post-treatment scheme served as a positive antiviral control. Cells were treated with VIP (10 nM), ANT (1000 nM), or a combination of both compounds following the pre-treatment or post-treatment protocols described above. Subsequently, cells were infected with HSV-1 at an MOI of 1 for 1 h, according to the corresponding experimental scheme, and maintained for 24 h in the presence of post-treatment or in Opti-MEM medium. At 24 h.p.i., cells were harvested and pelleted by centrifugation at 1000× *g* for 5 min at 4 °C. Cell lysates were then prepared, and the effect of the treatments on viral infection was assessed by detecting gB expression through Western blot analysis in six independent experiments.

### 4.7. RT-qPCR and Differential Gene Expression Analysis from Publicly Available RNA-Seq Data

Total RNA was extracted from brains and trigeminal ganglia at days 4 and 30 post-HSV-1 infection using TRIzol reagent (Thermo Fisher Scientific), following the manufacturer’s instructions. For SH-SY5Y cells, cell pellets were resuspended in TRIzol and extracted, following the manufacturer’s instructions. Here, 200 ng of RNA was used for each reaction using TaqMan RNA-to-CtTM 1-step (Thermo Fischer Scientific) and TaqMan probes for the detection of mouse *NPY* (Mm01410146_m1), *VIP* (Mm00660234_m1), and human *NPY* (Hs00173470_m1) and *VIP* (Hs00175021_m1). Reactions were run on the StepOnePlusTM Real-Time PCR System (Applied Biosystems, Foster City, CA, USA) with the following cycling conditions: one cycle at 65 °C for 15 min and 95 °C for 10 min, followed by 40 cycles at 95 °C for 15 s and 60 °C for 1 min. For relative abundance determination, β-actin (Mm02619580_g1 for mouse samples and Hs99999903_m1 for neuroblastoma human cells) was used as the housekeeping gene using the 2^−∆∆Ct^ method. Moreover, the differential expression of *VIP* and *NPY* was assessed using the publicly available raw counts and normalized counts per million (CPM) matrices from brainstem samples (GSE168799) [29]. The differential expression was computed with DESeq2. A single model (~group) was fit across all six sample groups (wt/mut × NI/HSV-LO/HSV-HI, n = 2–4 per group), and pairwise contrasts were extracted via Wald tests, including genotype comparisons within each infection condition, infection-vs.-NI comparisons within each genotype, and pooled infected-vs.-NI and high-vs.-low responder comparisons.

### 4.8. Western Blotting

Cell pellets were lysed in ice-cold RIPA buffer (Pierce™, Thermo Fisher Scientific, CAT #89900) containing Halt™ 100X protease inhibitor (Thermo Fisher Scientific, CAT #78440) and sonicated for 1 min. Following lysis, cells were centrifuged at 12,000× *g* for 15 min, and supernatants were collected for storage at −80 °C. Prior to use, protein extracts were quantified using the Pierce™ BCA Protein Assay (Thermo Fisher Scientific, CAT #23227). Here, 30 µg of each sample was prepared for SDS-PAGE protein separation. Samples were loaded onto 12% polyacrylamide gels and run in Tris-Glycine buffer until needed. Proteins were transferred to 0.45 μm nitrocellulose membranes for 105 min at 150 mA at 4 °C (NPY1R, VIPR1 and glycoprotein B) or to a 0.2 μm methanol-activated PVDF membrane for 70 min at 400 mA at 4 °C (NPY and VIP). Membranes were blocked with 5% skim milk in TBS (NPY1R, VIPR1 and glycoprotein B) or 3% Bovine Serum Albumin (NPY and VIP). Membranes were incubated with anti-NPY1R (1:1000, Novus Biologicals, Centennial, CO, USA, CAT #NBP1-80987), anti-VIP1R (1:1000, Novus Biologicals, CAT #NBP3-33097), anti-gB (1:1000; Virusys, Taneytown, MD, USA, CAT #HA025-1), anti-NPY (1:1000, Cell Signaling Technology, Danvers, MA, USA, Neuropeptide Y #D7Y5A), anti-VIP (1:1000, Novus Biologicals VIP-BSA Free-Rabbit polyclonal antibody #NBP3-12900), and anti-Actin (1:4000, GeneScript, Piscataway, NJ, USA, CAT #A00730) overnight at 4 °C. Following primary antibody incubation, membranes were washed with TBS-T (0.1%) three times for 5 min. HRP-conjugated secondary antibodies were incubated for 1 h at room temperature (RT) (1:5000, Cell Signaling Technology CAT #7074 Anti-rabbit; 1:5000, Anti-goat, R&D Systems, Minneapolis, MN, USA, CAT #HAF017; and 1:20,000, Anti-mouse, Bio-Rad, Hercules, CA, USA, CAT #1706516). After incubation, the membranes were washed with TBS-T (0.01%) three times for 5 min. Following membrane washing, membranes were revealed using Immobilon^®^ Forte Western HRP Substrate (Millipore, Burlington, MA, USA, CAT #WBLUF0500) in an iBright 1500 Imaging System (Thermo Fisher Scientific) for NPY1R, VIPR1 and gB or Clarity TM Western ECL Substrate (BIO-RAD, Hercules, CA, USA) and revealed in a LI-COR MODEL 2800 (LICORbio, Lincoln, NE, USA) for NPY and VIP.

### 4.9. Statistical Analyses

The means and SEM are presented as summary statistics. One-way ANOVA was conducted when 3 or more groups were compared, with Tukey’s, Dunnett’s or Bonferroni’s multiple-comparison post hoc test or unpaired Student’s *t*-test when 2 groups were compared. To analyze the body weight and HSE score in mice, as well as receptor expression in *in vitro* experiments over time, two-way ANOVA with mixed effects was performed, followed by a post hoc Dunnett’s test against the healthy control. Survival rates were represented using a Kaplan–Meier curve. All statistical analyses were performed using GraphPad Prism 10 software (GraphPad Software, La Jolla, CA, USA). *p* < 0.05 was considered significant. Significance from GSE168799 data was defined as Benjamini–Hochberg-adjusted *p* (*padj*) and nominal *p*-value < 0.05.

## 5. Conclusions

Taken together, this study reveals significant differences in the expression of these molecules between two mouse strains with divergent disease severity outcomes, which may be associated with HSE severity and chronic damage, warranting further evaluation. It is the first report to evaluate the modulation of neuropeptide expression during HSV-1 brain infection, which appears to be largely dependent on both host susceptibility and viral neurovirulence. The higher neuropeptide levels during latency in resistant versus susceptible animals and the concurrent upregulation of neuropeptide mRNA and downregulation of NPY1R protein in human neuronal cells lays the foundations for future studies aimed at determining whether NPY and VIP could contribute to neuroimmune regulation during HSV-1 infection.

## Data Availability

The original contributions presented in this study are included in the article/Supplementary Materials. Further inquiries can be directed to the corresponding author(s).

## Supplementary Materials

The following supporting information can be downloaded at https://www.mdpi.com/article/10.3390/ijms27156735/s1.
