# Supplementary material for: HSV-1 Infection Differentially Modulates NPY and VIP Neuropeptide Expression in the Mouse Brain and in Human Neuronal Cells
Source: Int J Mol Sci. 2026 Jul 28;27(15):6735. doi: 10.3390/ijms27156735 (PMC13466417; doi:10.3390/ijms27156735)
Supplement: Supplementary file 1 [file ijms-27-06735-s001.zip › ijms-4358487-supplementary.pdf]

HSV-1 Infection Differentially Modulates NPY and VIP Neuropeptide Expression in the Mouse Brain and in Human Neuronal Cells

Javier Carbone-Schellman [1,2], Nicolás Sales-Salinas [1,2], Rodrigo Reyes-Ramírez [2,3], Benjamín Rodríguez [1,2], Patricia Pereira-Sánchez [2,3], Gisella Vásquez-Canales [4], José A. Jara [4], Alfredo Molina-Berríos [4], Boris Rebollledo-Jaramillo[5], Alexis M. Kalergis [2,3,6], Pablo A. González [2,3] and Luisa F. Duarte [1,2,*]

[1]Centro de Medicina Regenerativa, Facultad de Medicina, Clínica Alemana, Universidad del Desarrollo, Santiago 7610717, Chile; javier.carbone@udd.cl (J.C.-S.); n.salessalinas@uandresbello.edu (N.S.-S.); benja.rodriguez.m@gmail.com (B.R.)

[2]Millennium Institute on Immunology and Immunotherapy, Santiago 8331150, Chile; rodrigoreyesr1992@gmail.com (R.R.-R.); patricia.pereira@uc.cl (P.P.-S.); akalergis@uc.cl (A.M.K.); pagonzam@uc.cl (P.A.G.)

[3]Facultad de Ciencias Biológicas, Pontificia Universidad Católica de Chile, Santiago 8330025, Chile

[4]Institute for Research in Dental Sciences, Faculty of Dentistry, Universidad de Chile, Santiago 8380544, Chile; gvasquez@odontologia.uchile.cl (G.V.-C.); jsandovalj@uchile.cl (J.A.J.); aemolina@u.uchile.cl (A.M.-B.)

[5]Rare Diseases Program, Center for Genetics and Genomics, Institute of Science and Innovation in Medicine, Facultad de Medicina, Clínica Alemana, Universidad del Desarrollo, Santiago 7610717, Chile; brebolledo@udd.cl (B.R.-J.)

[6]Departamento de Endocrinología, Facultad de Medicina, Pontificia Universidad Católica de Chile, Santiago 8330023, Chile

*Correspondence: lduarte@udd.cl

SUPPLEMENTARY FIGURES

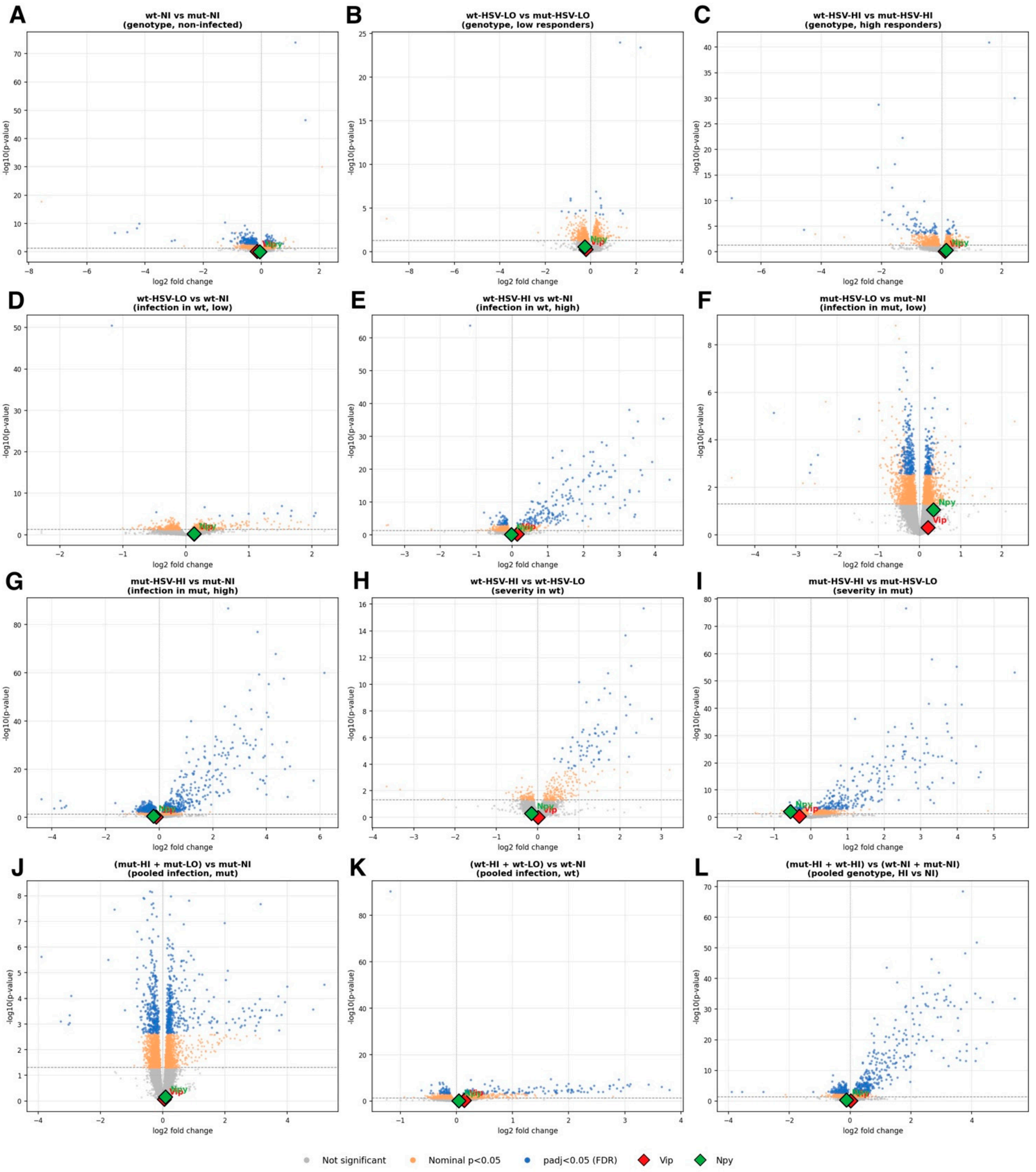

Supplementary Figure S1. Reanalysis of the public brainstem RNA-seq dataset GSE168799 showing Npy and Vip expression in wild-type and Rel^{C307X} C57BL/6 mice following HSV-1 infection. Publicly available raw counts and normalized counts per million (CPM) matrices from GSE168799 (Mancini et

al., 2021) were reanalyzed to assess Vip and NPY expression. Samples correspond to brainstems of wild-type ($Rel^{+/+}$, "wt") and c-Rel-mutant (Rel^{C307X}, "mut") female C57BL/6 mice, collected at day 5 post-intranasal HSV-1 infection along with non-infected controls (NI, wt: n = 4; mut: n = 4); infected animals were further stratified into low- (HSV-LO, wt: n = 2; mut: n = 3) and high-responder (HSV-HI, wt: n = 3; mut: n = 3) subgroups based on viral transcript load. (A–L) Volcano plots (log2 fold change vs. −log10 nominal p-value) for pairwise contrasts described above: genotype effects at matched infection status (A–C), infection effects within each genotype (D–G), severity effects within each genotype (H, I), pooled infection effects within each genotype (J, K), and the pooled infection-status effect irrespective of genotype (L). Genes are colored by significance (not significant, grey; nominal $p < 0.05$, orange; *p* adjusted values (padj) < 0.05, blue), with VIP (red diamond) and NPY (green diamond) individually highlighted. A significant nominal *p value* in NPY mRNA expression was detected only in mut-HSV-HI vs mut-HSV-LO (fold change (FC) = 0.680, $\log_2 FC = -0.556$, nominal $p = 0.0083$, padj = 0.2275). Neither NPY nor VIP reached significance after multiple-testing correction (all *padj* > 0.05). Detailed sample size per comparison, fold changes, nominal *p-values*, and *padj* values are reported in Supplementary Table 1.

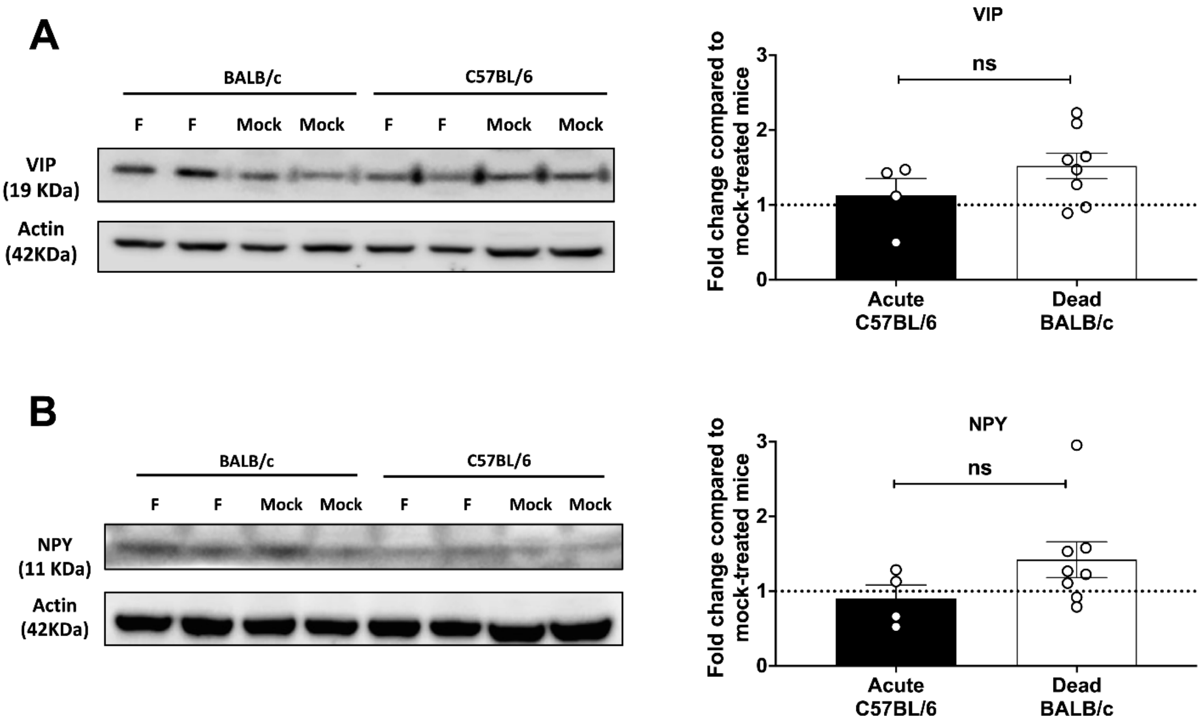

Supplementary Figure S2. Comparison of VIP and NPY protein expression in brain homogenates from BALB/c animals that succumbed to infection and C57BL/6 mice during acute HSV-1 infection.. Western blot analysis of VIP (A) and NPY (B) protein expression in whole brain homogenates from C57BL/6 mice at the acute phase of infection (4 dpi; "Acute C57BL/6") and BALB/c mice that succumbed to HSV-1 encephalitis ("Dead BALB/c"), compared to their respective mock-treated controls. Representative blots are shown (left panels); β-actin (42 kDa) was used as a loading control. Quantification is expressed as fold change relative to mock-treated mice of the same strain (right panels; dotted line = 1). Both neuropeptides showed a trend toward increased protein levels in Dead BALB/c mice compared to Acute C57BL/6 mice, but differences did not reach statistical significance (ns; Mann-Whitney test). F = HSV-1-infected; Mock = mock-treated. Data represent mean ± SEM.

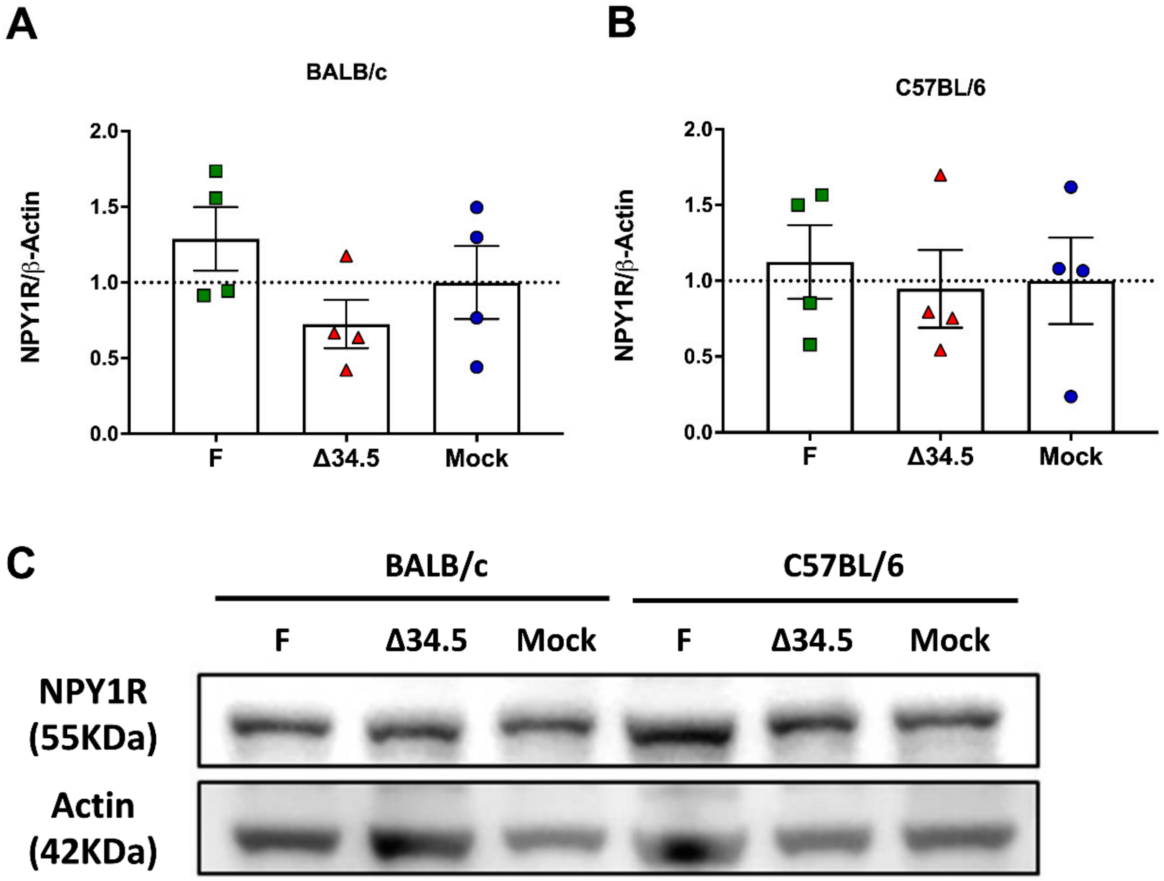

Supplementary Figure S3. NPY1R protein expression remains unchanged in the brains of BALB/c and C57BL/6 mice during acute HSV-1 infection. Western blot analysis of NPY1R expression in whole brain homogenates from BALB/c (A) and C57BL/6 (B) mice infected with wild-type HSV-1 strain F (F), the neuroattenuated ICP34.5-null mutant (Δ34.5), or mock-infected controls, all evaluated at 4 days post-infection. A representative blot is shown in panel (C); β-actin (42 kDa) was used as a loading control. NPY1R protein levels are expressed as the ratio to β-actin and normalized to the mock group (dotted line = 1). One-way ANOVA with Tukey's post-hoc test was performed. No statistically significant differences in NPY1R protein levels were observed among the three experimental groups in either mouse strain. Data represent mean ± SEM; individual data points are shown.

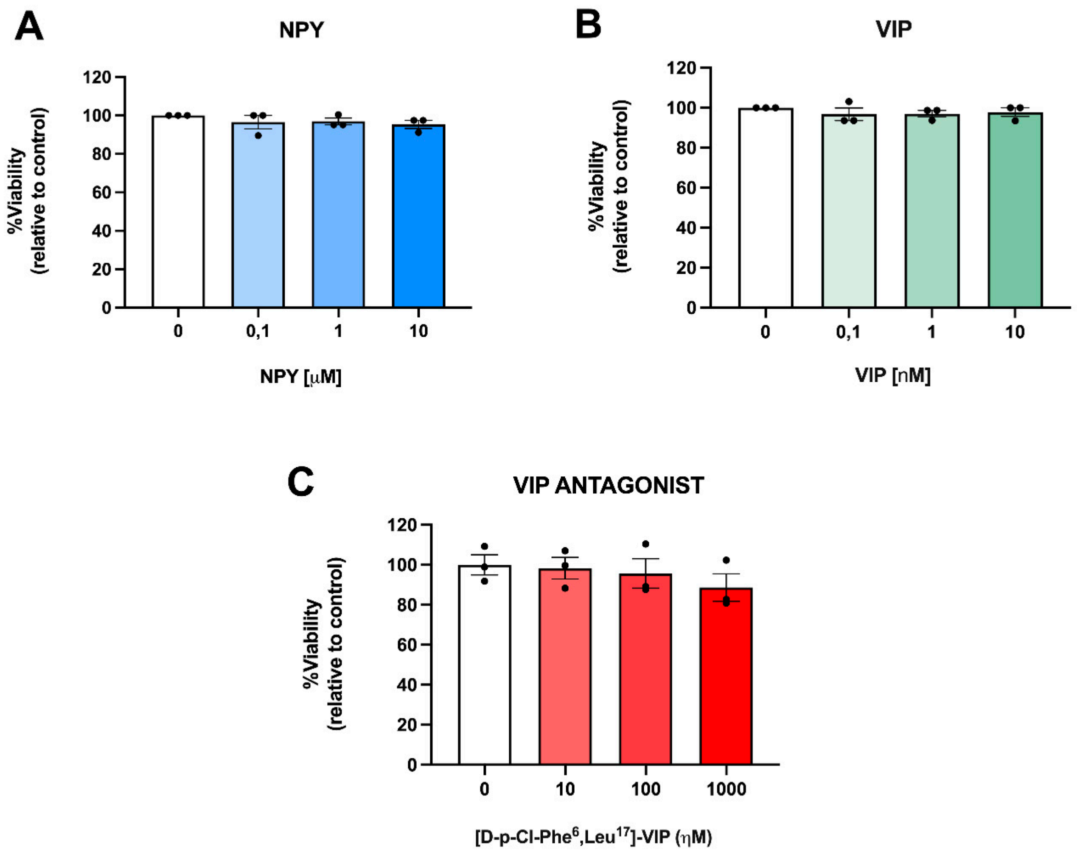

Supplementary Figure S4. Effect of NPY, VIP, and a VIP receptor antagonist on SH-SY5Y cell viability. H-SY5Y cells were treated with increasing concentrations of NPY (0.1, 1, and 10 µM) (A) , VIP (0.1, 1, and 10 nM) (B), or the VIP receptor antagonist [D-p-Cl-Phe6,Leu17]-VIP (10, 100, and 1000 nM) (C) for 24 h. Cell viability was assessed using the resazurin reduction (AlamarBlue™) assay and is expressed as the percentage of viable cells relative to untreated controls. Data represent the mean ± SEM of three independent experiments. Statistical significance was determined by one-way ANOVA followed by Dunnett's multiple-comparisons test against the untreated control.

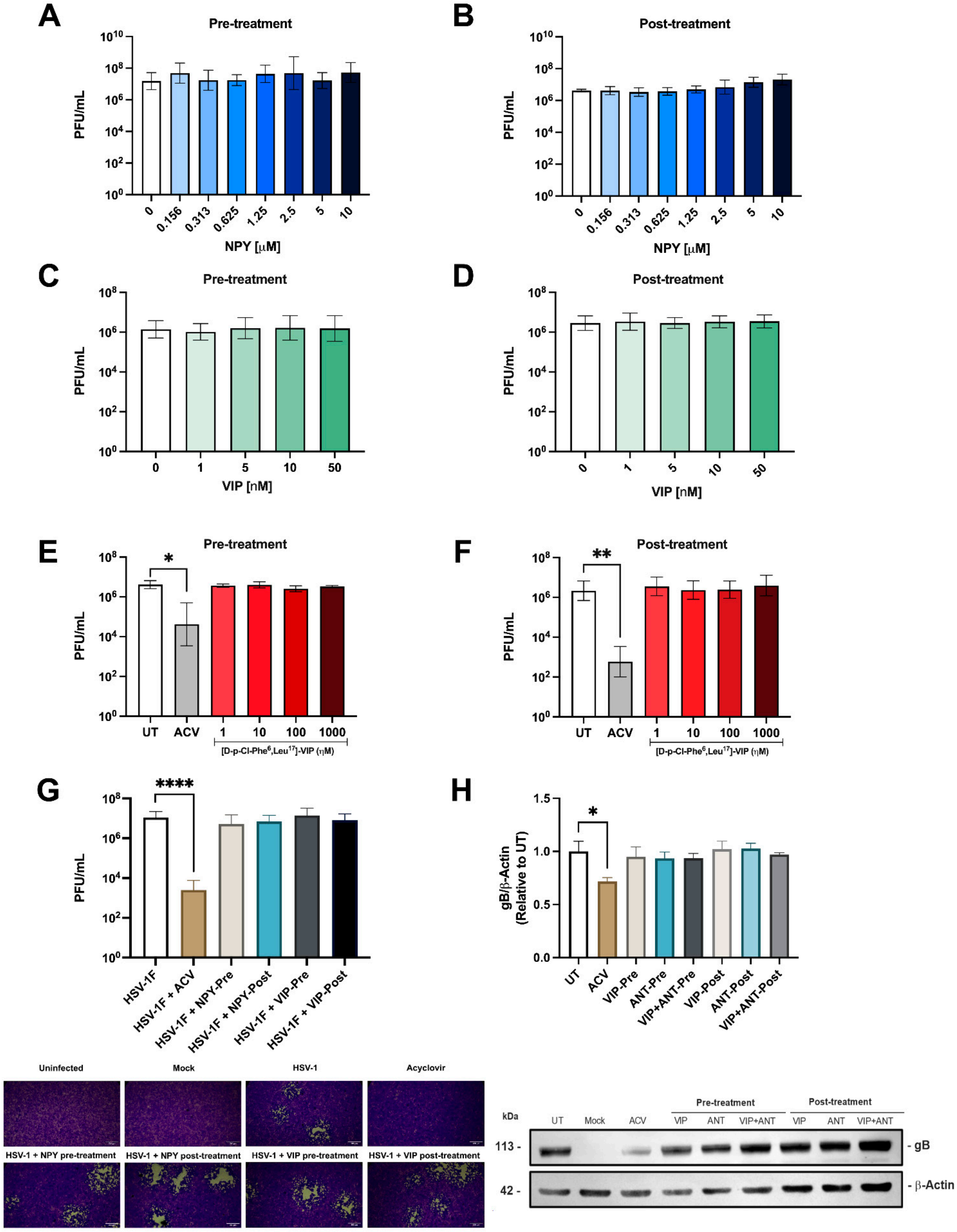

A
Pre-treatment
PFU/mL
0
0.156
0.313
0.625
1.25
2.5
5
10
NPY [μM]
B
Post-treatment
PFU/mL
NPY [μM]
C
Pre-treatment
PFU/mL
VIP [nM]
D
Post-treatment
PFU/mL
VIP [nM]
E
Pre-treatment
PFU/mL
UT
ACV
1
10
100
1000
[D-p-Cl-Phe6,Leu17]-VIP (ηM)
F
Post-treatment
PFU/mL
G
PFU/mL
HSV-1F
HSV-1F + ACV
HSV-1F + NPY-Pre
HSV-1F + NPY-Post
HSV-1F + VIP-Pre
HSV-1F + VIP-Post
H
gB/β-Actin (Relative to UT)
UT
ACV
VIP-Pre
ANT-Pre
VIP+ANT-Pre
VIP-Post
ANT-Post
VIP+ANT-Post
Uninfected
Mock
HSV-1
Acyclovir
HSV-1 + NPY pre-treatment
HSV-1 + NPY post-treatment
HSV-1 + VIP pre-treatment
HSV-1 + VIP post-treatment
kDa
Pre-treatment
Post-treatment
UT
Mock
ACV
VIP
ANT
VIP+ANT
113 -
42 -
- gB
- β-Actin

Supplementary Figure S5. Effect of NPY, VIP, and VIP-antagonist on HSV-1 replication in SH-SY5Y cells. The antiviral activity of NPY and VIP was assessed by quantifying the release of infectious viral particles from culture supernatants collected at 24 h post-infection (h.p.i.) following pre-treatment or post-treatment experimental schemes. (A–B) PFU/mL in SH-SY5Y cells treated with NPY across a concentration range of 1.25–10 µM under pre-treatment (A) or post-treatment (B) conditions (n=3). (C–D) PFU/mL in cells treated with VIP across a concentration range of 1-50 ηM under pre-treatment (C) or post-treatment (D) conditions (n=3). (E–F) Effect of the VIP antagonist [D-p-Cl-Phe^{6},Leu^{17}]-VIP (ANT; 1–1000 nM) on viral replication under pre-treatment (E) or post-treatment (F) conditions (n=3). Acyclovir (ACV) was included as a positive antiviral control; untreated infected cells (UT) served as baseline. (G) PFU/mL in cells treated with NPY (1 µM, pre- or post-treatment) or VIP (10 nM, pre- or post-treatment), compared to UT and ACV-treated cells (n=4). (H) Effect of VIP (10 nM), ANT (1000 nM), and VIP+ANT combination on HSV-1 glycoprotein B (gB) expression assessed by western blot at 24 h.p.i., expressed as gB/β-actin ratio relative to UT cells. ACV (post-treatment) was used as a positive antiviral control; mock-infected cells were included as a negative control (n=6). Representative crystal violet-stained Vero cell monolayers used for plaque quantification are shown (bottom left, scale bar 200 µm), and a representative western blot for gB and β-actin is shown (bottom right). Statistical comparisons were performed with One-way ANOVA with Dunnet´s post-hoc test against untreated group (* $p < 0.05$, ** $p < 0.01$, **** $p < 0.0001$). Data represent mean ± SEM.

Supplementary Table S1. Differential expression analysis of *NPY* an *VIP* across all pairwise comparisons in the GSE168799 brainstem RNA-seq dataset showed in Supplementary Figure 1.

Gene	Comparison	Group A	Group B	n (A)	n (B)	Mean CPM A	Mean CPM B	log2FC (A vs B)	Fold change	p-value	padj (FDR)	Nominal p<0.05	padj<0.05
Npy	wt-NI vs mut-NI (genotype, non-infected)	wt-NI	mut-NI	4	4	6,349	6,534	-0,047	0,968	0,7975	0,9388	No	No
Npy	wt-HSV-LO vs mut-HSV-LO (genotype, low responders)	wt-HSV-LO	mut-HSV-LO	2	3	6,897	8,271	-0,257	0,837	0,2615	0,846	No	No
Npy	wt-HSV-HI vs mut-HSV-HI (genotype, high responders)	wt-HSV-HI	mut-HSV-HI	3	3	6,284	5,648	0,152	1,111	0,4756	0,9377	No	No
Npy	wt-HSV-LO vs wt-NI (infection in wt, low)	wt-HSV-LO	wt-NI	2	4	6,897	6,349	0,127	1,092	0,5617	0,9999	No	No
Npy	wt-HSV-HI vs wt-NI (infection in wt, high)	wt-HSV-HI	wt-NI	3	4	6,284	6,349	-0,021	0,985	0,9146	0,9997	No	No
Npy	mut-HSV-LO vs mut-NI (infection in mut, low)	mut-HSV-LO	mut-NI	3	4	8,271	6,534	0,336	1,262	0,0869		No	No
Npy	mut-HSV-HI vs mut-NI (infection in mut, high)	mut-HSV-HI	mut-NI	3	4	5,648	6,534	-0,22	0,859	0,2738	0,5366	No	No
Npy	wt-HSV-HI vs wt-HSV-LO (severity in wt)	wt-HSV-HI	wt-HSV-LO	3	2	6,284	6,897	-0,148	0,903	0,5216	0,9999	No	No
Npy	mut-HSV-HI vs mut-HSV-LO (severity in mut)	mut-HSV-HI	mut-HSV-LO	3	3	5,648	8,271	-0,556	0,68	0,0083	0,2275	Yes	No
Npy	(mut-HSV-HI + mut-HSV-LO) vs mut-NI	mut-infected	mut-NI	6	4	6,959	6,534	0,085	1,06	0,6943	0,861	No	No
Npy	(wt-HSV-HI + wt-HSV-LO) vs wt-NI	wt-infected	wt-NI	5	4	6,529	6,349	0,044	1,031	0,7702	1	No	No
Npy	(mut-HSV-HI + wt-HSV-HI) vs (wt-NI + mut-NI)	HI-pooled	NI-pooled	6	8	5,966	6,441	-0,118	0,921	0,4076	0,7213	No	No
Vip	wt-NI vs mut-NI (genotype, non-infected)	wt-NI	mut-NI	4	4	3,15	3,452	-0,146	0,904	0,5978		No	No
Vip	wt-HSV-LO vs mut-HSV-LO (genotype, low responders)	wt-HSV-LO	mut-HSV-LO	2	3	3,429	4,001	-0,219	0,859	0,5283	0,9384	No	No
Vip	wt-HSV-HI vs mut-HSV-HI (genotype, high responders)	wt-HSV-HI	mut-HSV-HI	3	3	3,482	3,225	0,105	1,075	0,7396	0,9783	No	No
Vip	wt-HSV-LO vs wt-NI (infection in wt, low)	wt-HSV-LO	wt-NI	2	4	3,429	3,15	0,13	1,094	0,6943	0,9999	No	No
Vip	wt-HSV-HI vs wt-NI (infection in wt, high)	wt-HSV-HI	wt-NI	3	4	3,482	3,15	0,141	1,103	0,6321	0,9997	No	No
Vip	mut-HSV-LO vs mut-NI (infection in mut, low)	mut-HSV-LO	mut-NI	3	4	4,001	3,452	0,203	1,151	0,4931		No	No
Vip	mut-HSV-HI vs mut-NI (infection in mut, high)	mut-HSV-HI	mut-NI	3	4	3,225	3,452	-0,11	0,927	0,7136	0,8649	No	No
Vip	wt-HSV-HI vs wt-HSV-LO (severity in wt)	wt-HSV-HI	wt-HSV-LO	3	2	3,482	3,429	0,011	1,008	0,974	0,9999	No	No
Vip	mut-HSV-HI vs mut-HSV-LO (severity in mut)	mut-HSV-HI	mut-HSV-LO	3	3	3,225	4,001	-0,312	0,805	0,3222	0,8136	No	No
Vip	(mut-HSV-HI + mut-HSV-LO) vs mut-NI	mut-infected	mut-NI	6	4	3,613	3,452	0,055	1,039	0,8413	0,9317	No	No
Vip	(wt-HSV-HI + wt-HSV-LO) vs wt-NI	wt-infected	wt-NI	5	4	3,461	3,15	0,14	1,102	0,5027	1	No	No
Vip	(mut-HSV-HI + wt-HSV-HI) vs (wt-NI + mut-NI)	HI-pooled	NI-pooled	6	8	3,353	3,301	0,016	1,011	0,9425	0,9831	No	No

wt, wild-type (Rel^{+}/$^{+}$); mut, c-Rel mutant (RelC307X); NI, non-infected; HSV, herpes simplex virus type 1-infected; LO, low viral load; HI, high viral load; n, number of animals per group; CPM, counts per million; log2FC, log$_2$ fold change; FDR, false discovery rate; padj, multiple-testing-adjusted *p*-value.
